# Statistics of Epilepsy in the Philadelphia Hospital, (Blockley)

**Published:** 1844-06-29

**Authors:** 


### STATISTICS OF EPILEPSY IN THE PHILADELPHIA HOSPITAL, (BLOCKLEY.)

WITH REMARKS, by M. M. WILSON, M. D., at the time Resident Physician,—now of Schenectady.

(Communicated by Professor DUNGLISON.)

*November*, 1841.

| | 1 | 2 | 3 | 4 | 5 | 6 | 7 | 8 | 9 | 10 | 11 | 12 | 13 | 14 | 15 | 16 | 17 | 18 | 19 | 20 | 21 | 22 | 23 | 24 | 25 | 26 | 27 | 28 | 29 | 30 | 31 |
|---|---|---|---|---|---|---|---|---|---|---|---|---|---|---|---|---|---|---|---|---|---|---|---|---|---|---|---|---|---|---|---|
| | | | | | moon 3 qr. | | | | | | | | new moon | | | | | | | | moon 1 qr. | | | | | | | full moon | | | |
| J. Gregg, æt. 27 | | | NO STATISTICS. | | | | | | | | | | | | | | | 16 | | | | | | | | 6 | — | 16 | | | |
| *J. Baily, 23 | | | | | | | | | | 1 | 1 | | | | 1 | | | | | 1 | | | 1 | | | — | | | | | |
| A. Laughlin, 38 | | | | | | | 4 | 1 | | | | | | | | | | | 1 | | | | | | 1 | | | | | 1 | |
| M. Fayville, 23 | | | | | | | | | | | | | | 2 | | | | | | | | | 1 | | | | | | | | |
| C. Howard, 35 | | | | | | | | | | | | | | | | | | | | | | | | | | | | | | | |
| M. McCan, 15 | | | | | | | 3 | 3 | | | | | | | | | | | | | | | | | | | | — | | | |
| M. Layton, 36 | | | | | | | | | | 1 | 1 | 1 | 1 | | | | 1 | | | | | | | | 1 | 1 | 1 | 1 | | | |
| M. Clark, 12 | | | | | | | | | | | | | | | | | | | | | | | | | | | = | ≡ | | | |

*December.*

| | 1 | 2 | 3 | 4 | 5 | 6 | 7 | 8 | 9 | 10 | 11 | 12 | 13 | 14 | 15 | 16 | 17 | 18 | 19 | 20 | 21 | 22 | 23 | 24 | 25 | 26 | 27 | 28 | 29 | 30 | 31 |
|---|---|---|---|---|---|---|---|---|---|---|---|---|---|---|---|---|---|---|---|---|---|---|---|---|---|---|---|---|---|---|---|
| | | | | | moon 3 qr. | | | | | | | new moon | | | | | | | | moon 1 qr. | | | | | | | | full moon | | | |
| J. Gregg, | 1 | 1 | | | | | 1 | 1 | | | | | | 3 | | | | | | | | 5 | | 3 | 6 | | | | | | 15 |
| A. Laughlin, | | 2 | | | | | | | cl | cl | c | cl | cl | | | | 1 | | 1 | | | | | 1 | | | | | | | |
| M. Fayville, | | | | | c | c | c | cl | c | c | | | | 1 | 3 | | | 10 | | | | | | | | | 2 | | | | |
| C. Howard, | | | | | | | | | | | | | | | | | | | | | | | | | | | | | | | |
| M. Layton, | | 1 | | | | | c2 | cl | cl | c | | | | | | | | | | | | | | | | | 1 | 1 | 2 | 3 | 2 |
| M. Clark. | | | | | | | | | | | | | | | | | | | | | | | | | 1 | | | | | | |

*January*, 1842.

| | 1 | 2 | 3 | 4 | 5 | 6 | 7 | 8 | 9 | 10 | 11 | 12 | 13 | 14 | 15 | 16 | 17 | 18 | 19 | 20 | 21 | 22 | 23 | 24 | 25 | 26 | 27 | 28 | 29 | 30 | 31 |
|---|---|---|---|---|---|---|---|---|---|---|---|---|---|---|---|---|---|---|---|---|---|---|---|---|---|---|---|---|---|---|---|
| | | | moon 3 qr. | | | | | | | | new moon | | | | | | | | moon 1 qr. | | | | | | | full moon | | | | | |
| J. Gregg, | 13 | 8 | 1 | — | — | | — | c | c | c | c | c | c | | — | | | | | | | | | — | — | | | | ≡ | | |
| A. Laughlin, | | 1 | | | | c | c | cl | c | 1 | | | | | 2 | | | | | | | | | | | | | | | | |
| M. Fayville, | | | | | | | | | c | c | c | c | c | c2 | | | | | | | | | | | | | | | 2 | | |
| C. Howard, | 13 | 4 | 2 | | | | | | | | | | | | | | | | | | | | c | c | c | c | | | | | |
| M. Layton, | 1 | | | | | | | | c | c | c | c | c | | | | | | | | | | | | | | | | | | |
| M. Clark, | | | | | | | | | | | | | | | | | | | | | | | | | 1 | | | | | | |

*February.*

| | 1 | 2 | 3 | 4 | 5 | 6 | 7 | 8 | 9 | 10 | 11 | 12 | 13 | 14 | 15 | 16 | 17 | 18 | 19 | 20 | 21 | 22 | 23 | 24 | 25 | 26 | 27 | 28 | 29 | 30 | 31 |
|---|---|---|---|---|---|---|---|---|---|---|---|---|---|---|---|---|---|---|---|---|---|---|---|---|---|---|---|---|---|---|---|
| | | moon 3 qr. | | | | | | | | new moon | | | | | | | | moon 1 qr. | | | | | | full moon | | | | | | | |
| J. Gregg, | | c | c | c | c | c | cl | 1 | | 24 | 2 | | | | | 1 | | | | | 7 | | | | | | 1 | | | | |
| A. Laughlin, | | 2 | | | | | | | 1 | | | | 1 | | | | | 1 | | | | | 1 | | | | | | | | |
| M. Fayville, | | | | | | c | c | c | c | | | 1 | | | | | | | | | | | | | | | | 4 | | | |
| C. Howard, | | | | | | | | | | | | | | | | | | | | | | | | | | | c | c3 | c | c | c |
| M. Layton, | | | | | | | | | | c | c3 | c | c | | | | | | | | | | | | | | | | | | |
| M. Clark, | | | | | | | | | | | | | 1 | | | | | | 2 | | | | | | | | | | | | |
| M. Ridey, æt. 29 | | | | | | | | | | | | | | | | | | | | | 2 | | | | | | | c | c | c | c |

*March.*

| | 1 | 2 | 3 | 4 | 5 | 6 | 7 | 8 | 9 | 10 | 11 | 12 | 13 | 14 | 15 | 16 | 17 | 18 | 19 | 20 | 21 | 22 | 23 | 24 | 25 | 26 | 27 | 28 | 29 | 30 | 31 |
|---|---|---|---|---|---|---|---|---|---|---|---|---|---|---|---|---|---|---|---|---|---|---|---|---|---|---|---|---|---|---|---|
| | | | moon 3 qr. | | | | | | | | | new moon | | | | | | | moon 1 qr. | | | | | | | full moon | | | | | |
| J. Gregg, | | | | | | | | 4 | | | 8 | 4 | | | 7 | 7 | 4 | | 1 | | 1 | 1 | | — | | — | | | | | — |
| A. Laughlin, | 1 | | | | 1 | | | | c | c2 | c | c | 1 | | | | 1 | | | | 2 | 1 | | 1 | | | | 2 | | | 1 |
| M. Fayville, | 3 | | | | | c | c | c | c | c | | | | | 1 | 2 | | | | | | | | | | | | | | 2 | |
| C. Howard, | | c | c | | | | | | | | | | | | | | | | | | | | | | | | | c | c | c | c |
| M. Layton, | | | | | c | c | c | c | c | | | | | | | | | | | | | | | | | | | | | | |
| M. Ridey, | | | | | c | c | c | c | c | | | 5 | 9 | | | | | | | | | | | | | | | | | | |
| †M. Spratt, æt. 40 | | | | | | | | | | | | | | | | | | | cl | c | c | c | c | c | c | | 1 | | | | |
| ‡M, Branninn, 39 | | | | | | | | | | | | | | | | | | | | | | | | | | | | | | | |

*April.*

| | 1 | 2 | 3 | 4 | 5 | 6 | 7 | 8 | 9 | 10 | 11 | 12 | 13 | 14 | 15 | 16 | 17 | 18 | 19 | 20 | 21 | 22 | 23 | 24 | 25 | 26 | 27 | 28 | 29 | 30 | 31 |
|---|---|---|---|---|---|---|---|---|---|---|---|---|---|---|---|---|---|---|---|---|---|---|---|---|---|---|---|---|---|---|---|
| | | | moon 3 qr. | | | | | | | new moon | | | | | | | | moon 1 qr. | | | | | | full moon | | | | | | | |
| J. Gregg, | | | | | | | | 15 | | 1 | 1 | — | | — | | | — | | — | | | | 1 | c | c | c | c | c | | | |
| A. Laughlin, | | c | c | c | cl | c | | | | | | 1 | | | | | | | | | | | | | | | | | | | |
| M. Fayville, | | | c | c | c | c | c | 1 | | | | | | | | | | | | | | | | | | | | | | | |
| C. Howard, | | | | | | | 6 | 1 | | | | | | | | | | | | | | | c | c | c | c | c | | | | |
| M. McCan, | | | | | | | 2 | 1 | | | | | | | | | | | | | | | | | | | 1 | 3 | 1 | | |
| §M. Layton, | | | | | | | | | | | | | | | | | | | | | | | | | | | | | | | |
| A. Spratt, | | | 1 | | | | | | | | | | | | | 1 | | | | | | 1 | | | | | | 1 | | | |
| ‖M. Brannin, | | | | | | | | | | | c | c | c | c | c | | | | | | | 1 | | 1 | | | | | | | |

*May.*

| | 1 | 2 | 3 | 4 | 5 | 6 | 7 | 8 | 9 | 10 | 11 | 12 | 13 | 14 | 15 | 16 | 17 | 18 | 19 | 20 | 21 | 22 | 23 | 24 | 25 | 26 | 27 | 28 | 29 | 30 | 31 |
|---|---|---|---|---|---|---|---|---|---|---|---|---|---|---|---|---|---|---|---|---|---|---|---|---|---|---|---|---|---|---|---|
| | | moon 3 qr. | | | | | | | | new moon | | | | | | | moon 1 qr. | | | | | | | full moon | | | | | | | |
| J. Gregg, | — | | — | — | | | | | | — | | | | | | | | | | | — | | | | | | | | | | |
| A. Laughlin, | c | c | cl | c | c | | | | | 1 | | | | 2 | | | | | | 2 | | | | | | | | | | | |
| M. Fayville, | c | c | c8 | c | c | | 1 | | | | | | | | | | | | | | | | | | | | | | | | |
| C. Howard, | | | | | | | | | | | | | | | | | | | | | | | | | 5 | 2 | | | | | |
| M. McCan, | | | | | | | | | | | | | | | | | | | | 4 | 1 | | | | | | | | | | |
| A. Spratt, | | 1 | | 1 | | | | | | | | | | 1 | 2 | | | | | | | | | | 1 | | | | | | |

* Died November 29th. † Entered the Ward 14th of March. ‡ Entered the Ward 14th of March.

§ Became insane, and was confined in the Lunatic Asylum. ‖ Left the house 25th of April.

c Catamenia present.

NOTE.—The figures opposite the patients' names show the number of convulsions they had on the day of the month indicated by the upper row of figures. The horizontal marks indicate the days on which the convulsions were incomplete—syncope. The quarters of the moon are written above the statistics of each month.

*History of the cases.*—J. Gregg has been subject to epilepsy, since she was fourteen years old. They come on without assignable cause. A feeling of dulness, or heaviness, generally precedes the attacks. Intelligence nearly lost; complexion dark; eyes and hair black; unmarried.

J. Baily is unmarried—no history.

Ann Laughlin—no history; intelligence almost lost. She has attacks of mania of a peculiar nature, occasionally. These attacks generally commence with venereal mania, followed in twenty-four hours by a conscientious horror of a fancied indulgence, which lasts for a day or two, and a violent religious mania succeeds, continuing sometimes for a week or two, when she again becomes sane. It is worthy of remark, that she at first complains of severe pain in the back part of the head, in the region where the phrenologists place amativeness. As the venereal mania leaves her, this pain passes off, and she then complains of pain on the side of the head where they place conscientiousness, and this is followed in like manner with pain on the top of the head, in the organ of veneration. She is unmarried. Dark complexion.

M. Fayville has been subject to epilepsy for three years. Was attacked suddenly, without assignable cause. Her health is generally good; mind much impaired; complexion dark.

C. Howard has been married for eleven years. She was attacked with epilepsy about a year before her marriage; she does not know what to attribute it to. She has three children. Pain in the head precedes the attack for a day or two. Intellect but little impaired.

M. McCan was first attacked four years ago. She attributes it to the ill-treatment of her employer. She has been troubled with incontinence of urine since she was seven years old. Now somewhat relieved by the use of blisters to the sacrum, and the internal use of tincture of cantharides.

M. Layton has been subject to epilepsy for seven years. She attributes it to interruption of the menstrual function. She is intemperate.

The other cases have nothing of interest attached to their history that could be ascertained.

*Remarks.*—The object of the above statistics was to discover what cause excited the paroxysm, in order to arrive at a more rational mode of treatment. It is an old notion, which, even at the present day, has its adherents, that epileptic convulsions are in some way connected with the phases of the moon. By reference to this table, it will be seen, that in these cases no connection whatever existed. Neither did they follow regular periods, although some of them, as Howard, were certain to be free from them for several weeks after an attack; nor were they, in any case, uniformly confined to the menstrual period. These observations were continued for nine months; the notes for June and July have by some means been mislaid; the result, however, was the same. From these statistics it is clear that the exciting cause must be looked for elsewhere than in lunar influence, periodicity and menstruation. After these facts had been satisfactorily ascertained, attention was directed to the food, and other sources of irritation and excitement immediately preceding the paroxysm; and these observations appeared to develope the exciting cause in most, if not all, the patients in the ward. A few hours after taking indigestible food, or an unusually full meal, they almost invariably had attacks of epilepsy. On the first day of January, the nurse gave them a feast, and a reference to the table will show that almost every patient in the ward had convulsions on that and the following day. Mental depression and undue excitement were almost invariably followed by a similar result. I have known several of them to be attacked with violent convulsions during an inflammatory address by a certain minister, who frequently came to the hospital to administer "spiritual consolation" to its inmates. This was so uniformly the consequence of the minister's preaching, that devotional exercises were prohibited in the ward. Two patients were very irritable, and were thrown into violent passion on trifling occasions, and convulsions generally followed.

In the treatment of these cases, almost every remedy that had been suggested by the profession had been tried by my predecessors, and with little or no benefit. Profiting by their experience, I made use of no therapeutical agents directed to epilepsy itself, but prescribed such medicine and regimen as were deemed proper for the improvement of the general health, and perhaps with some benefit. But as no previous statistics had been preserved, it was impossible to decide whether the number of convulsions were diminished or not. In the opinion of the nurse they were materially so. The nitrate of silver was administered with apparent benefit in a few cases where the digestive organs were impaired. This remedy is often prescribed empirically, and we must believe frequently with success.

